# Effect of sugammadex on chest radiographic abnormality in the early postoperative period after video-assisted thoracoscopic lobectomy

**DOI:** 10.3906/sag-2001-26

**Published:** 2020-08-26

**Authors:** Dong Kyu LEE, Sung Wook KANG, Hyun Koo KIM, Hyo Sung KIM, Heezoo KIM

**Affiliations:** 1 Department of Anesthesiology and Pain Medicine, Korea University Guro Hospital, Korea University College of Medicine, Seoul Republic of Korea; 2 Department of Anesthesiology and Pain Medicine, Nowon Chuck Hospital, Seoul Republic of Korea; 3 Thoracic and Cardiovascular Surgery, Korea University Guro Hospital, Korea University College of Medicine, Seoul Republic of Korea

**Keywords:** Thoracic radiography, postoperative complications, sugammadex, anticholinesterases, video-assisted thoracic surgery

## Abstract

**Background/aim:**

Sugammadex, which offsets the effects of neuromuscular blocking agents (NMBs), has advantages over traditional reversal agents like pyridostigmine, as it enables fast and reliable recovery from neuromuscular blockade. This study compared the incidence of early postoperative chest radiographic abnormalities (CRA) between sugammadex (group S) and pyridostigmine (group P) following video-assisted thoracoscopic (VAT) lobectomy for lung cancer.

**Materials and methods:**

We performed a retrospective cohort analysis by reviewing the medical records of patients who underwent VAT lobectomy at a single university medical center. We defined the early postoperative CRA as a characteristic appearance on chest radiograph up to 2 days after surgery. Arterial blood gas analysis (ABGA), surgical time, anaesthesia time, extubation time, and the total dose of rocuronium were analysed. Postoperative nausea and vomiting (PONV) and pain scores were observed until 2 days after surgery.

**Results:**

A total of 257 patients underwent VAT lobectomy during the study period; 159 were included in the final analysis. Ninety patients received sugammadex while 69 received pyridostigmine. The incidence of early postoperative atelectasis was significantly lower in group S than in group P (26.7%, 95% CI: 17.5%‒35.8% and 43.5%, 95% CI: 31.8%‒55.2%, respectively, P = 0.013). The median dose of rocuronium was higher in group S than in group P (120 mg vs. 90 mg, P < 0.001). ABGA, extubation time, and PONV were similar in both groups.

**Conclusion:**

Sugammadex decreased the incidence of CRA in the early postoperative period despite higher NMB consumption.

## 1. Introduction

Video-assisted thoracoscopic surgery (VATS) enables rapid postoperative recovery and reduces the incidence of postoperative pain and other complications compared to conventional thoracotomy [1–3]. Single-port VATS with a minimal incision of 3–5 cm has been advocated as the surgical method for lung lobectomy, a currently available technique in lung cancer surgery [4–7]. It offers several advantages over 3-port VATS, including less postoperative pain and a better cosmetic result [8]. However, this surgical procedure requires delicate techniques within a limited surgical field, a profound neuromuscular block is essential and requires a higher dose of NMB use. Consequently, it is important to be sure about enhanced recovery from NMBs to reduce the risk of respiratory complications [9,10]. 

The reversal agent with anticholinesterase plays a limited role when a high dose of muscle relaxant is used [11–14]. Sugammadex binds to steroidal nondepolarizing muscle relaxants in a 1:1 ratio, completely antagonizes its neuromuscular blocking effect, and is excreted through the kidneys as inactivated form. Depending on its dose, sugammadex effectively reverses neuromuscular blocking agents induced by rocuronium even in the presence of profound neuromuscular block [15].

Therefore, we hypothesised that using sugammadex as a reversal agent could enhance the ability of pulmonary rehabilitation compared to using anticholinesterase. To prove this hypothesis, we compared early postoperative respiratory abnormalities that appeared on chest radiography between using 2 different reversal agents after the single-port VAT lobectomy.

## 2. Materials and methods

### 2.1. Study design and setting

This study was approved by the institutional review board (KUGH15280-001) and we performed a single-center retrospective cohort study in a university teaching hospital. Between January 2013 and August 2015, 257 patients who underwent a single-port VAT lobectomy at the Korea University Medical Center Guro Hospital (Seoul, Republic of Korea) were screened for eligibility. Since our institution has started to use sugammadex in late 2013, we included the patients who underwent single-port VAT lobectomy for about 1 year before and after 2014. The recruited patients had been managed by 3 anesthesiologists who were specialists in thoracic anesthesia and the operation was performed by 1 thoracic surgeon. Using the internal hospital code corresponding to a single-port VAT lobectomy, the hospital medical record management team provided the data from the electronic medical system. 

### 2.2. Patient population

**Figure 1:**
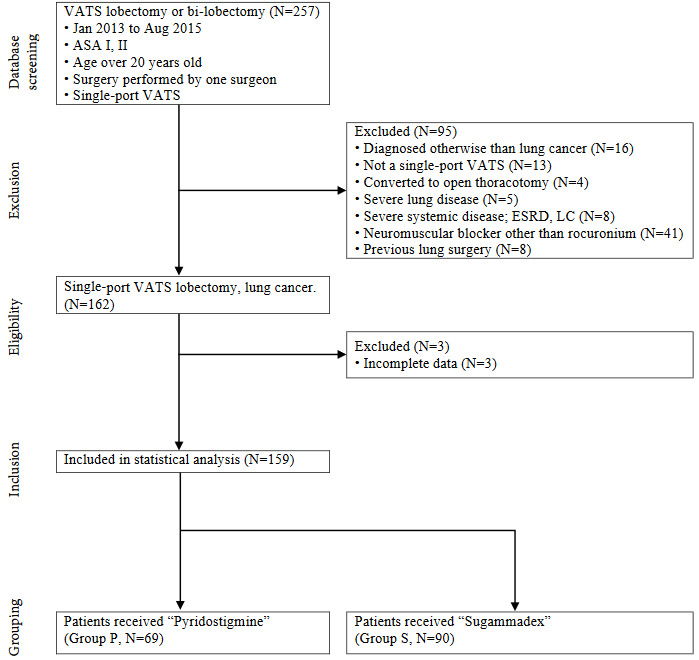
Flo w diagram showing patient inclusion, exclusion, and st atistical analysis. All patient data were collected by medical record review. VATS: video-assisted thoracic surgery; ASA: American Society of Anesthesiologists: ASA is the name of organization, using American style typing. Physical status classifications; ESRD: end-stage renal disease; LC: liver cirrhosis.

We included patients who met the following criteria: aged over 19 years, elective lung lobectomy or bilobectomy using a single-port VATS, and American Society of Anesthesiologists (ASA) physical status class I or II. We excluded patients with previous history of thoracic surgery, surgery other than single-port VATS, surgery other than lung cancers, preexisting respiratory/abdominal muscle weakness or neuromuscular diseases, congestive heart failure, asthma, chronic obstructive pulmonary disease or emphysema (other than stage I), other chronic pulmonary diseases that could affect postoperative lung function (for example: pneumonia caused from bacteria or virus, lung abscess), end-stage renal disease on dialysis, diagnosed with Child-Pugh classification A or over hepatic impairment, and the use of neuromuscular blocking agents other than rocuronium during anesthesia, the case of conversion to thoracotomy during surgery, and patients with unconfirmed chest radiography pre or postoperatively. Of the 257 patients reviewed, we excluded 98 patients in accordance with the exclusion criteria as detailed in Figure 1. One hundred and fifty-nine patients were included in the final analysis. Patients who received sugammadex at the end of anesthesia were classified as group S (n = 90), and those who received pyridostigmine were classified as group P (n = 69). 

Anesthesia was induced using propofol or pentothal sodium and maintained using sevoflurane or desflurane with N2O. With invasive monitoring for arterial and central venous pressure, one-lung ventilation (OLV) was achieved using a double-lumen endotracheal tube or a single-lumen endotracheal tube with a bronchial blocker. Continuous infusion of remifentanil or bolus of fentanyl was administrated when required. Rocuronium (EsmeronTM Inj., N.V. Organon, Oss, the Netherlands) was the sole agent used for facilitating endotracheal intubation and muscle relaxation during anesthesia. A minimal intraoperative fluid management strategy was applied to all patients. Mechanical ventilation was applied during anesthesia as follows: before OLV, a tidal volume (TV) of 8 mL/kg, respiration rate (RR) of 12 cycles/min, inspiration to expiration ratio (I:E) of 1:2, with or without 5~10 cmH2O positive end-expiratory pressure (PEEP), and 50% FiO2 with N2O; during OLV, a TV of 6~7 mL/kg, RR of 15~16 cycles/min, I:E of 1:2, with or without 5~10 cmH2O PEEP, and 100% FiO2. The alveolar recruitment maneuver was performed in all patients at the end of surgery and 2-lung ventilation was restored. To reverse the effects of NMBs, sugammadex (BridionTM Inj., N.V. Organon, the Netherlands) or pyridostigmine (PyrinulTM Inj., Myungmoon Pharm., Seoul, Republic of Korea) and glycopyrrolate (MobinulTM Inj., Myungmoon Pharm., Seoul, Republic of Korea) were administered. After emergence from anesthesia, the patient was extubated and transferred to the intensive care unit (ICU). Standardized postoperative management was applied to all patients including semi-Fowler’s position, active coughing and deep breathing encouragement, periodic position changes for bronchial toilet, chest percussions when required, minimizing oxygen administration as possible, and pain control principles.

### 2.3. Data collection

Provided medical records were reviewed separately by 2 investigators and validated by 1 other investigator. If there was a discordant between the collected data, three investigators looked up the provided medical records and a decision was made after in-depth discussion. Information on age, sex, height, body weight, ASA physical status class, smoking history, medical histories of hypertension, diabetes, asthma, and chronic obstructive lung disease, including emphysema, preoperative pulmonary function tests (forced expiratory volume in 1 s [FEV1], forced vital capacity [FVC], and diffusing capacity of the lung for carbon monoxide [DLCO]), and the types of surgery were collected. Preoperative pulmonary symptoms including cough, sputum, and dyspnea were also investigated. During surgery, anesthetic factors were also gathered, including durations of anesthesia and surgery, providing 1-lung ventilation, intraoperative mechanical ventilator settings, arterial blood gas analysis (ABGA) results, total dose of rocuronium used, whether or not sugammadex was used and its dose, and total fluid administered, including crystalloid and colloid. Using blood products during the operation was also counted separately. Postoperative chest radiography findings from the day of operation to 2 postoperative days were also investigated. Postoperative pain scores on a 10-point numerical rating scale (NRS) were used in the surgical ICU and general ward. Reported postoperative nausea and vomiting (PONV) were also counted as followed from the nursing care records.

### 2.4. Outcome measurement and definitions

The primary outcome was the incidence of early postoperative chest radiographic abnormalities (CRA). Early postoperative CRA on aeration was defined as the chest radiologists’ report during postoperative 2 days, including pneumonic infiltrations, lobar, segmental, and subsegmental atelectasis confirmed by a certified radiologist on postoperative chest radiography. If chest radiography report was not available even though the patient had taken chest radiography, we excluded the case. Other chest radiographic findings also followed from the report of the chest radiologist. Pneumonia was defined when the patient presented pneumonic infiltration on chest radiography with clinical symptoms or laboratory findings indicating pneumonia, including a fever over 38 °C, purulent sputum that was not reported before surgery, leukocytosis that was not explained by another cause except lung infection. With clinical findings, pleural effusion and hemothorax were also recorded when they were presented simultaneously on the chest radiography report and medical records. The secondary outcome was identifying the parameters that could be a cause of early postoperative CRA among the collected variables. 

### 2.5. Statistical analysis

We used IBM SPSS Statistics for Windows (Version 23.0, IBM Corp., Armonk, NY, USA, 2015) and R software 3.5.2 (R Development Core Team, Vienna, Austria, 2018). All data were coded with numerical values and titles of variables were also named with coded characters to blind the statistician. Before statistical analysis, missing data analysis was performed. For the variable that had missing values over 5% of cases, we inspected those missing values that occurred at random; then imputation was applied with the univariate imputation method of classification and regression trees, maximal iteration was set at 250, the number of multiple imputations was 5 [16]. We assessed the imputed values with an overimputation diagnostic plot [17]. Otherwise, we performed a complete case analysis for variables with missing values of less than 5%. Intraoperative ABGA checked approximately a half an hour after establishing 1-lung ventilation for thoracoscopic surgery presented a 9.7% missing rate. The missing pattern of this variable was not related to the patients’ characteristics; we performed missing value imputation as planned. After imputation, cross-validation was adequate.

For continuous variables, a normality test was performed with the Shapiro–Wilk test. If a variable violated the normality, nonparametric methods were applied. Student’s t-test, Mann–Whitney U test, one-way ANOVA with 1 repeated factor, chi-square test, or Fisher’s exact test were performed depending on the type of variables. In the one-way ANOVA with 1 repeated factor, a sphericity test was performed using the Mauchly’s method. If the sphericity assumption was violated, Greenhouse–Geisser correction was performed. The ANOVA results were interpreted by verifying the interaction between variables. The primary outcome variable, early postoperative CRA, was evaluated with a z-test to compare the estimated incidence rate. Multiple logistic regression was performed to investigate the possible effects of demographic data on early postoperative CRA. The variables were determined by a backward stepwise selection method, tested with log-likelihood estimation. Correlation analysis of the selected variables was performed to evaluate the interaction and collinearity. The final model was tested with Hosmer-Lemeshow statistics for the goodness-of-fit and a visual check of Cook’s distance. Bootstrapping was used with 1000 numbers of simple sampling methods. The fitted model was presented with an odds ratio (OR) and its 95% CI.

Results were presented as mean ± SD, median (IQR) or number (percentile) for the measured data, mean (95% confidence intervals [CI]), or number (ratio [95% CI]) for the estimated values. Statistical results were presented with 2-sided P values and corresponding effect sizes. P value of less than 0.05 was considered statistically significant.

## 3. Results

All preoperative parameters were not statistically different between the 2 groups, except diabetes mellitus prevalence. The prevalence of diabetes mellitus in group S was 20.0%, higher than in group P with small effect size (5.8%, P = 0.011, Crémer’s V = 0.20, Table 1). Among intraoperative parameters, there were no statistical differences between groups with respect to N2O used, fluid administered volume, anesthesia time, operation time, extubation time, and units of transfusion (Table 2). Group S received a higher dose of rocuronium (120.0 mg [100.0–140.0 mg]) compared to group P with moderate effect size (90.0 mg [70.0–107.5 mg], P < 0.001, r = 0.55). The median dose of pyridostigmine in group P was 10 mg (10–10 mg), 5 patients in group P received 15 mg of pyridostigmine for NMBs reversal, otherwise, 10 mg of pyridostigmine were administered. Patients in group S received 2.08 mg/kg (2.01–2.20 mg/kg), ranging from 1.38 to 3.77 mg/kg. Expired tidal volume during 1-lung ventilation was not different between groups (339.6 ± 52.8 mL vs. 350.0 ± 60.8 mL for groups P and S, respectively, P = 0.223, Cohen’s d = 0.20). The median scores of maximum pain measured using NRS were not different between groups (3.0 [3.0–5.0] vs. 3.0 [3.0–4.0]) for groups P and S, respectively, P = 0.734, r = 0.03). PONV incidence was not statistically different. 

**Table 1:** Preoperative patient characteristics.

Parameters		Group P (n = 69)	Group S (n = 90)	Mean difference	P value	Effect size
Age (years)		59.7 ± 9.2	59.5 ± 9.5	0.2 (–3–3.5)	0.888	0.03
Body mass index (kg/m2)		23.14 ± 2.99	23.16 ± 3.23	–0.02 (–1.11–1.07)	0.973	0.01
No. of female		19 (27.5%)	30 (33.3%)	-	0.490	0.06
Current smoking		15 (21.7%)	16 (17.8%)	-	0.551	0.05
Hypertension		26 (37.7%)	33 (36.7%)	-	>0.99	0.01
Diabetes mellitus		4 (5.8%)	18 (20.0%)	-	0.011*	0.20
Chronic lung disease		5 (7.2%)	3 (3.3%)	-	0.295	0.09
Preoperative chest symptoms	Cough	11 (15.9%)	11 (12.2%)	-	0.644	0.05
	Sputum	9 (13.0%)	10 (11.1%)	-	0.807	0.03
	Dyspnoea	2 (2.9%)	2 (2.2%)	-	>0.99	0.02
FVC%		88.9 ± 13.9	88.5 ± 10.9	0.3 (–4.1–4.7)	0.882	0.03
FEV_1_%		85 ± 16.8	84.3 ± 13.2	0.7 (–4.6–6)	0.784	0.05
DLCO%		87.6 ± 19.6	81.8 ± 17.8	5.8 (–0.8–12.3)	0.084	0.31
Site of operation	RUL	16 (23.2%)	21 (23.3%)	-	0.553	0.21
	RML	5 (7.2%)	4 (4.4%)			
	RLL	18 (26.1%)	23 (25.6%)			
	RUL&RML	0 (0%)	3 (3.3%)			
	RUL&RLL	0 (0%)	2 (2.2%)			
	RML&RLL	5 (7.2%)	3 (3.3%)			
	LUL	12 (17.4%)	21 (23.3%)			
	LLL	13 (18.8%)	13 (14.4%)			

Data are presented as mean ± standard deviation or number of patients (percentile). Mean differences are presented with a 95% confidence interval. Group P: patients who received pyridostigmine as the reversal agent. Group S: patients who received sugammadex as the reversal agent. FVC%: percentile of functional vital capacity; FEV1%: percentile of forced expiratory volume in 1 s; RUL: right upper lobe; RML right middle lobe; RLL: right lower lobe; LUL: left upper lobe; LLL: left lower lobe. The effect size was presented with Cohen’s d and Crémer’s V for student’s t-test and chi-squared test, respectively. *: indicates a 2-sided P value <0.05.

**Table 2:** Intraoperative and postoperative parameters.

Parameters		Group P (n = 69)	Group S (n = 90)	P value	Effect size
Amount of rocuronium (mg)		90.0 (70.0–107.5)	120.0 (100.0–140.0)	<0.001*	0.55
Amount of pyridostigmine (mg/kg)		0.167 (0.153–0.189)	0 (0–0)	-	-
Amount of sugammadex (mg/kg)		0 (0–0)	2.08 (2.01–2.20)	-	-
N2O used during two-lung ventilation		39 (56.5)	63 (70.0)	0.079	0.14
N2O used during one-lung ventilation		6 (8.7)	8 (8.9)	0.966	0.00
Anaesthesia time (min)		245.0 (222.5–300.0)	225.0 (220.0–310.0)	0.535	0.05
Operation time (min)		180.0 (149.5–236.5)	186.5 (150.8–230.5)	0.999	0.00
One-lung ventilation time (min)		160 (130–202.5)	163 (132.5–215)	0.756	0.03
Extubation time (min)		8.0 (5.0–15.0)	7.0 (5.0–12.0)	0.266	0.20
Fluid administered (mL)		1050.0 (800.0–1337.5)	950.0 (700.0–1475.0)	0.769	0.02
Red blood cell transfusion		8 (11.6%)	6 (6.7%)	0.398	0.09
Expired tidal volume during two-lung ventilation (mL)		500 (500–600)	500 (450–550)	0.251	0.09
Respiratory rate during two-lung ventilation (cycles/min)		12 (12–12)	12 (12–13)	0.067	0.15
Expired tidal volume during OLV (mL)		339.6 ± 52.8	350.0 ± 60.8	0.223	0.20
Respiratory rate during OLV (cycles/min)		15 (15–16)	15 (15–16)	0.086	0.14
Maximal pain score		3.0 (3.0–5.0)	3.0 (3.0–4.0)	0.734	0.03
PONV		8 (11.6%)	4 (5.5%)	0.235	0.11
pH	30 min after OLV	7.425 ± 0.052	7.446 ± 0.054	<0.001*	0.083
	Immediate post operation	7.357 ± 0.039	7.381 ± 0.041		
	POD0 night	7.403 ± 0.053	7.408 ± 0.033		
	POD1 morning	7.407 ± 0.028	7.415 ± 0.027		
PO2	30 min after OLV	203.6 ± 95.4	228.9 ± 104.8	0.044*	0.026
	Immediate post operation	149.6 ± 52.1	124.1 ± 39.3		
	POD0 night	183.6 ± 52.9	151 ± 49.9		
	POD1 morning	162 ± 47.4	142.3 ± 45.4		
PCO2	30 min after OLV	41.6 ± 9.4	38.5 ± 6.4	0.003*	0.057
	Immediate post operation	44.9 ± 5.9	42.3 ± 4.6		
	POD0 night	39.6 ± 5.4	39.7 ± 4.4		
	POD1 morning	39.9 ± 5.7	38.6 ± 5.4		

Data are presented as median (IQR), mean ± SD, or number (percentile). Group P: patients who received pyridostigmine as the reversal agent. Group S: patients who received sugammadex as the reversal agent. OLV: 1-lung ventilation; POD0: Postoperative day 0; POD1: postoperative day 1; POD2: postoperative day 2. Extubation time measured from the anaesthetics-off to the time of endotracheal tube extubation. The administered fluid volume present is the summed value of crystalloid and colloid, except the volume of blood products used. The maximal units of transfused red blood cells were 3. Maximal pain score is maximal NRS pain score measured from POD0 to POD2. PONV: number of patients with postoperative nausea and vomiting episode. The effect size was presented with a common effect size r for Mann–Whitney U test and Crémer’s V for chi-squared or Fisher’s exact tests, partial η2 for one-way ANOVA with one repeated factor. *

**Figure 2:**
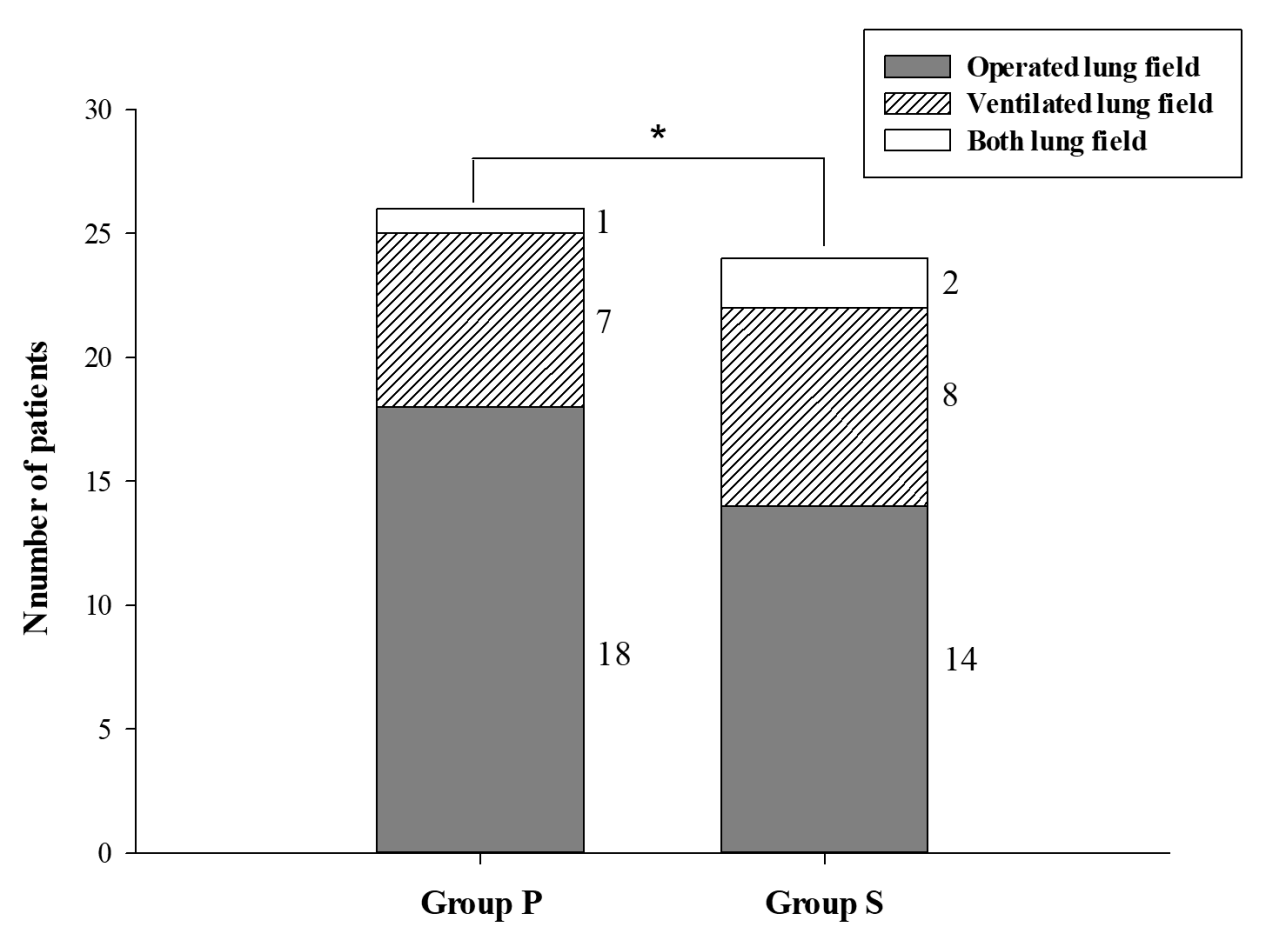
The incidence of early postoperative chest radiographic abnormalities. Numbers printed at the left side of each bar indicate the lung field where abnormalities occurred; its legend is presented. *: 2-sided P < 0.050 with z-test. Incidence was 43.5% vs. 26.7% for groups P and S, respectively, P = 0.013, Cohen’s h = 0.35. There was no significant difference between groups on the lung fields where abnormalities occurred (P = 0.359, Crémer’s V = 0.14, Fisher’s exact test).

Overall incidence of early postoperative CRA was 29.4% (95% CI: 22.6%–25.2%, Table 3). The incidence in group S was moderately lower compared to group P (26.7% [95% CI: 17.5%–35.8%] and 43.5% [95% CI: 31.8%–55.2%] in groups S and P, respectively, P = 0.013, Cohen’s h = 0.35, Figure 2). These were mainly observed at operated lung side in both groups, not different between groups (P = 0.359, Crémer’s V = 0.14). Pleural effusion was developed similarly in both groups (42.0% vs. 36.7% for groups P and S, respectively, P = 0.245, Crémer’s V = 0.11). Hemothorax, when counted separately from others, was observed only in group P (4.3% vs. 0.0% for groups P and S, respectively, P = 0.080, Crémer’s V = 0.11). No patients presented overt pneumonia with symptoms and signs. 

**Table 3:** Postoperative chest radiographic abnormalities.

Parameters		Group P (n = 69)	Group S (n = 90)	P value	Effect size
Chest radiographic changes on aeration		30 (43.5%)	24 (26.7%)	0.013*	0.35
Changes developed on	Operated lung	18 (26.1%)	14 (15.6%)	0.359	0.14
	Ventilated lung	7 (10.1%)	8 (8.9%)		
	Both lungs	1 (1.4%)	2 (2.2%)		
Pleural effusion		29 (42.0%)	33 (36.7%)	0.245	0.11
Haemothorax		3 (4.3%)	0 (0.0%)	0.080	0.16
Pneumonia with overt symptoms and signs		0 (0.0%)	0 (0.0%)	-	-

Data are expressed as the number of patients (percentile). Group P: patients who received pyridostigmine as the reversal agent. Group S: patients who received sugammadex as the reversal agent. Postoperative pain was counted when the measured score was over 3 using the numerical rating scale. Postoperative nausea and vomiting (PONV) were counted as follows from the nursing care records and antiemetics administration history. *: indicates 2-sided P value < 0.05. Effect size:

 Parameters related to early postoperative CRA were evaluated through binary logistic regression (Table 4). In the case of using sugammadex for neuromuscular blocker reversal, CRA decreased significantly (odds ratio = 0.273, 95% CI: 0.112–0.669, P = 0.005). As age increment by 1 year, the odds of CRA have increased 1.078 folds (95% CI: 1.021–1.137, P = 0.006). Higher BMI had a significant OR for CRA (1.161, 95% CI: 1.017–1.324, P = 0.027). Female sex, current smoking, hypertension, DLCO%, and PONV were included in the final model, and they presented insignificant results after bootstrapping.

**Table 4:** The odds ratio of parameters related to the occurrence of early postoperative chest radiographic changes on aeration.

Parameters (coding)	Univariate		Multivariate	
	OR (95%CI)	P value	OR (95% CI)	P value
Group (sugammadex use)	0.209 (0.073–0.601)	0.004	0.273 (0.112–0.669)	0.005
Sex (female)	2.512 (0.865–7.294)	0.09	1.976 (0.753–5.186)	0.166
Age (years)	1.064 (1.000–1.132)	0.051	1.078 (1.021–1.137)	0.006
Body mass index (kg/m2)	1.165 (1.010–1.345)	0.037	1.161 (1.017–1.324)	0.027
ASA classification (II)	2.327 (0.270–20.091)	0.442	-	-
Smoking (current)	2.603 (0.747–9.070)	0.133	2.811 (0.883–8.950)	0.080
Hypertension	0.338 (0.113–1.007)	0.051	0.401 (0.153–1.054)	0.064
Diabetes mellitus	1.429 (0.391–5.229)	0.59	-	-
Chronic lung disease	0.986 (0.136 – 7.158)	0.989	-	-
Preoperative cough	0.705 (0.151–3.282)	0.656	-	-
Preoperative dyspnoea	1.849 (0.360–9.505)	0.462	-	-
Preoperative dyspnoea	1.872 (0.168–20.872)	0.61	-	-
FVC%	0.992 (0.947–1.040)	0.749	-	-
FEV1%	1.002 (0.961 – 1.044)	0.931	-	-
DLCO%	1.030 (1.001–1.060)	0.046	1.025 (1.000– 1.051)	0.054
Intraoperative fluid administration (mL)	1.000 (0.999–1.001)	0.966	-	-
Allogenic red blood cell transfusion	2.452 (0.505–11.906)	0.266	-	-
Operation time (min)	0.999 (0.990–1.008)	0.806	-	-
PONV	0.111 (0.015–0.838)	0.033	0.189 (0.032–1.121)	0.067
Pain (over 3 NRS points)	0.532 (0.203–1.394)	0.199	-	-
Model summary	–2 Log likelihood = 135.70Overall correct classification: 64.6%		–2 Log likelihood = 139.763Overall correct classification: 67.7%	

The exponential value of regression coefficients (odds ratio, OR) are presented with their 95% confidence intervals. Multivariate binary logistic regression with backward stepwise selection method, tested with loglikelihood estimation. *: indicates a 2-sided P value <0.050. Bootstrapped results are presented for multivariate analysis.

Perioperative PaO2 showed similar changes in both groups but there were statistical differences between them (P = 0.044, partial η2 = 0.026, Table 2). However, the effect was small, and all measured values were in clinically normal oxygenation level. pH and PaCO2 were also statistically different between groups, all observed values remained within clinically insignificant range, and presented negligible effect size (P < 0.001, partial η2 = 0.083 for pH, P = 0.003, partial η2 = 0.057 for PaCO2). 

## 4. Discussion

To our knowledge, this is the first study on the effect of sugammadex on early postoperative CRA after single-port VAT lobectomy carried out with profound neuromuscular block. The incidence of CRA in the early postoperative period decreased more with the use of sugammadex than with pyridostigmine. Sugammadex resulted in enhanced recovery from the profound neuromuscular block, facilitating active deep breathing immediately after emergence from anesthesia; this might be beneficial in preventing early postoperative atelectasis. The effects of decreased incidence of CRA in the early postoperative period were considerable with a small OR of 0.273 compared to using anticholinesterase. This was true even when the patients with sugammadex received a higher dose of NMBs. The patients also maintained adequate respiration, not one patient presented significant CO2 retention postoperatively. Maintained normal PCO2 range during the early postoperative period also refers indirectly that patients have not experienced ventilation problems, including pathologic bronchial obstruction from secretions and inadequate respiratory muscle power [10].

Atelectasis is one of the most common postoperative respiratory complications after thoracic surgery [18]. Atelectasis could be the cause of hypoxemia in the early postoperative period and can be prevented with several simple measures, including chest physiotherapy, spontaneous deep breathing, and coughing. Copious secretions, pain, and respiratory muscle weakness are the common causes of atelectasis [19]. According to the severity of atelectasis, various clinical symptoms such as respiratory distress and hypoxemia could occur [20]. Atelectasis-induced respiratory distress and hypoxemia usually develop immediately after emergence from general anesthesia and could worsen until the postoperative day (POD) 2 [21]. Subsegmental atelectasis could not be associated with clinical symptoms from fever to hypoxemia [22]; it is common after thoracic surgery and does not respond to bronchoscope intervention [23]. To prevent these pathologic problems, preoperative exercise and postoperative respiratory rehabilitation, including deep breathing, active coughing, and expectoration are essential [24]. In our study, the use of sugammadex facilitated deep breathing, coughing, and expectoration due to an enhanced reversal of the effects of NMBs, thereby lowering the incidence of CRA. 

Our results could be a preceding finding for other positive prognostic outcomes of sugammadex use in various surgical settings [25–27]. Compared to previous reports on postoperative pulmonary complications [3,27], we eliminated multiple factors that could have resulted in postoperative respiratory complications, including patients with moderate-grade systemic disease and those who underwent complicated surgeries or intraoperative conversion to thoracotomy. Preoperative lung function in both study groups was similar and not so compromised. Intraoperative fluid management was based on minimal volume administration, and both groups underwent identical surgeries. Surgical and anesthesia times were also similar in both groups. Intraoperative mechanical ventilation was also performed according to the standardized OLV management protocol. Also, VAT lobectomy enhances early recovery [3] and the incidence of respiratory complications in the early postoperative period is between 15%–20% after VAT lobectomy [28]. In this situation, decreased incidence of CRA in patients that received sugammadex to reverse the effects of NMBs could be a promising point. Patients with relatively low risk of postoperative pulmonary complications could present various CRA that are related to postoperative pulmonary complications, and using sugammadex had the potential of reducing pulmonary complication risk even when the patients were compliant with pre and postoperative treatment to prevent complications.

Dose-dependent prolonged NMB increases the risk of postoperative respiratory complications [29]. In this study, the major difference between the groups was the total dose of rocuronium administered during anesthesia. In group S, the anesthesiologists tended to use a more liberal dose of rocuronium to induce profound neuromuscular block until the end of the surgery, expecting rapid and complete recovery from the neuromuscular block by using sugammadex. The duration of anesthesia was not different between groups, although a relatively large dose of rocuronium was used in group S. This finding suggests that sugammadex administration resulted in more rapid recovery from profound neuromuscular block compared to pyridostigmine. The number of patients with diabetes mellitus was higher in group S than in group P (5.8% vs. 20.0%, P = 0.011, Crémer’s V = 0.20). Diabetes mellitus is a known single risk factor for postoperative pulmonary complications [10]. Despite this finding, the incidence of CRA was low in group S, and the estimated multivariate logistic regression model did not include diabetes mellitus as a significant independent factor.

In our study, 5 patients developed postoperative pneumonic infiltration on chest radiography in the first 2 days after surgery. Among them, 4 patients had pneumonic infiltrates preoperatively, suspected to be secondary to cancer; all these lesions occurred at the operated lung side without any presenting symptoms. Only 1 patient had new-onset pneumonic infiltrates in the postoperative period. This patient had a history of right-sided chest wall trauma and recurrent pleural effusion. During the right upper lobectomy, extensive pleurolysis was performed for widespread pleural adhesions. Postoperatively, this patient developed dyspnea and right-sided consolidation. On POD 2, the bronchoscopic bronchial toilet was performed, followed by antibiotic therapy for 1 month. Following this, the patient was discharged without further complications. Hemothorax occurred in 3 patients, all in group P; the correlation coefficient with the use of sugammadex was 0.157, indicating a nonlinear relationship. 

Our study has a number of limitations. First, the development of respiratory complications was evaluated only until POD 2. However, as the half-life of rocuronium is 2 h, respiratory complications that occurred after POD 2 may not be related to the use of NMB. Secondly, sugammadex was introduced in our hospital in November 2013. Thus, most patients who underwent surgery after November 2013 were included in group S. However, single-port VATS lobectomy had been in active use since 2012, and the surgical technique was identical during the entire study period [7]. Therefore, the bias related to the inclusion of patients after November 2013 had minimal effect on the final analysis. Instead, we limited the patients receiving VAT lobectomy to between January 2013 and August 2015 only. By doing this, we could get a more homogenous cohort. Thirdly, most parameters that are already known as risk factors of postoperative pulmonary complications were similar between the groups and not significant in the multivariate logistic regression. In this study, most patients with high risk were excluded and investigated about CRA during early postoperative periods. These changes could either progress to overt pulmonary complications or not progress. It is an important finding that these changes could be prevented effectively with sugammadex, even in relatively healthy patients. Forth, the record about positive end-expiratory pressure (PEEP) during 1-lung ventilation was not clear in our medical records. PEEP has a considerable effect on atelectasis formation. Our hospital is a training institution; we have a protocolized training process for thoracic anesthesia, and in this protocol, the stepwise recruitment procedure should be supplied to all patients at the end of 1-lung ventilation. Uncertain medical recordings about PEEP could be a significant bias for our results; the recruitment performed at the end of surgery could abolish this bias. Fifth, the records about neuromuscular monitoring were not included in this study. During the study period, we could not find a consistent record of neuromuscular monitoring during anesthesia. Presuming from our institutional practice at that time, 2 mg/kg sugammadex were administered after confirmation of diaphragmatic movement; fortunately, we had not experienced inadequate recovery from neuromuscular blockade. No patient experienced postoperative hypoxemia required for mechanical ventilation or reintubation. Only 1 patient who received relatively extensive surgery due to previous trauma history suffered an unusual pulmonary complication. These could be possible considering the characteristics of included patients who were relatively healthy without preoperative pulmonary problems. Even in this population, sugammadex had superior effects on developing early postoperative ventilation problems. 

In conclusion, sugammadex use is associated with a lower incidence of early postoperative CRA after VAT lobectomy when compared to pyridostigmine. Sugammadex enables rapid and complete reversal of neuromuscular blockade via promoting active deep breathing, coughing, and expectoration, which prevents early postoperative ventilation abnormalities.

## Acknowledgement

This research did not receive any specific grant from funding agencies in the public, commercial, or not-for-profit sectors. The authors have read the STROBE statement – a checklist of items, and the manuscript was both written and revised according to the STROBE statement checklist of items. There is the registration of the Human Research Ethics Committee **-** Institutional Review Board of Korea University Guro Hospital Clinical Trial Centre, Reference No. (KUGH15280-001), approved on 10 December 2015.

## Informed Consent

As this was a retrospective study with de-identified data, we obtained a waiver of patient consent from the Institutional Review Board of Korea University Guro Hospital Clinical Trial Centre.
